# Assessing Midwives' Knowledge and Practice in Neonatal Resuscitation: Gaps and Transfer of Knowledge to Reduce Mortality

**DOI:** 10.1155/2024/6636506

**Published:** 2024-07-11

**Authors:** Mohamed Abdualgafar Osman Mohamedsharif, Isra Bdraldein Salih Mohammed, Abubaker A. Mohamedsharif

**Affiliations:** ^1^ Department of Pediatrics Faculty of Medicine Omdurman Islamic University, Khartoum, Sudan; ^2^ Anatomy Department Faculty of Medicine University of Khartoum, Khartoum, Sudan

## Abstract

**Introduction:**

The neonatal period is a crucial time for the survival, growth, and development of newborns. Despite advances in medical science, neonatal mortality rates remain a significant public health issue, and midwives play a critical role in reducing neonatal deaths through the use of evidence-based practices and appropriate neonatal resuscitation techniques. However, studies have shown that healthcare workers, including midwives, may not possess adequate knowledge in neonatal resuscitation, leading to adverse outcomes. This study aims to explore the current state of neonatal care and the role of midwives in neonatal resuscitation, with a focus on training and the transfer of knowledge into practice. It is essential to assess the level of knowledge of midwives in neonatal resuscitation and their ability to transfer this knowledge into action to reduce neonatal mortality rates.

**Objectives:**

The objective of this study was to assess the level of knowledge and practice of midwives in neonatal resuscitation, identify gaps in their knowledge and practice, and evaluate their ability to transfer this knowledge into action to reduce neonatal mortality rates.

**Methods:**

This study is a cross-sectional, descriptive study conducted in six governmental teaching hospitals located in Khartoum city, with a total sample size of 57 midwives who work in the labor and operation rooms of the hospitals. The questionnaire comprised sections on sociodemographics (5 questions), knowledge assessment (14 questions), and neonatal resuscitation practices (21 questions). The sampling technique used was total coverage.

**Result:**

This study included 57 female participants, primarily aged 51–60 years with a one-year diploma level of education. Of those surveyed, 49.1% performed neonatal resuscitation weekly. Participants demonstrated strong knowledge and practice in preparing for birth, but some gaps were identified in equipment usage and identification band placement. Resuscitation skills were generally lacking, with poor performance in ambo bagging and chest compression.

**Conclusion:**

This study on Sudanese midwives' neonatal resuscitation knowledge and practices reveals room for improvement in equipment use, identification, and resuscitation skills. Demographic factors affect CPR knowledge and practice.

## 1. Introduction

The neonatal period, which extends from birth to the first 28 days of life, is a critical time for the survival, growth, and development of newborns [1]. Immediate care of newborns after birth is crucial for their well-being. Good care during pregnancy, labor, and delivery is the first step in ensuring good newborn care. However, unsafe practices and behaviors related to the immediate care of newborns still exist, leading to adverse outcomes [2]. Despite advances in medical science, neonatal mortality rates have either stagnated or continued to rise in many countries [1]. Midwives play a vital role in reducing neonatal mortality, and training them in neonatal resuscitation is provided through various programs [3]. However, effective transfer of knowledge about neonatal resuscitation into practice is not easy, and there are gaps in the application of these skills. Rapid assessment can generally identify newborns who require resuscitation [4]. This study aims to explore the current state of neonatal care and the role of midwives in neonatal resuscitation with a focus on training and transfer of knowledge into practice.

Neonatal mortality remains a global concern and a significant public health issue. Healthcare providers, especially midwives, play a critical role in preventing neonatal deaths through evidence-based practice and appropriate neonatal resuscitation techniques [5]. However, studies have shown that healthcare workers, including midwives, may not have adequate knowledge in neonatal resuscitation [6, 7]. This lack of knowledge can lead to an increase in neonatal deaths worldwide, especially due to birth asphyxia [8]. Therefore, it is essential to assess the level of knowledge of midwives in neonatal resuscitation and their ability to transfer this knowledge into action to reduce neonatal mortality rates.

The study conducted among midwives in neonatal resuscitation yielded insightful findings regarding the knowledge and practices of these healthcare professionals in Khartoum, Sudan. Despite the presence of adequate knowledge and adherence to certain practices, areas requiring improvement were identified, such as the need for enhanced equipment utilization, attention to identification band details, and improvement in ambo bagging and chest compression skills. In addition, the study revealed the impact of demographic and experiential factors on CPR knowledge and practice proficiency. These results underscore the importance of targeted CPR training programs tailored to address specific factors influencing neonatal resuscitation quality, ultimately aiming to improve maternal and child healthcare outcomes in Sudan.

## 2. Materials and Methods

### 2.1. Study Design and Setting

This study was a cross-sectional, descriptive study conducted in six governmental teaching hospitals located in Khartoum city. The research was carried out over a period of ten months, from October 2019 to July 2020.

### 2.2. Study Population

The research involved all midwives employed in the labor and operation rooms of the teaching hospitals in Khartoum city, regardless of their full-time or part-time status. This encompassed a total of six governmental teaching hospitals. Midwives involved in obstetric care were included, while those working in other departments such as village midwives and sister midwives were excluded. Additionally, midwives working in private hospitals in Khartoum city and those on vacation were also excluded from the study, along with individuals who declined to participate.

### 2.3. Sampling

The sample size for the study was determined to be 57 midwives, which represented the total number of midwives in the six governmental teaching hospitals in Khartoum city. To obtain the sample, total coverage was used as the sampling technique.

### 2.4. Data Collection

Data was gathered through an interviewer-administered questionnaire, which was meticulously crafted to evaluate midwives' knowledge, practices, and observational skills concerning neonatal resuscitation. The comprehensive 40-question survey encompassed various sections focusing on sociodemographic details, knowledge assessment, and practical applications related to neonatal resuscitation.

The questionnaire design included 5 questions dedicated to gathering essential background information on the midwives, 14 questions aimed at assessing their comprehension of neonatal resuscitation topics, and 21 questions exploring their hands-on implementation of resuscitation techniques and protocols.

To ensure the questionnaire's reliability and validity, a pilot study was conducted to fine-tune its content and structure. In addition, the questionnaire underwent scrutiny by a panel of experts to confirm its appropriateness for assessing midwives' knowledge and practices in neonatal resuscitation.

Scoring and analysis of the questionnaire responses were meticulously carried out, with the knowledge section scores based on the accuracy of responses and the practices section scores reflecting the proficiency in applying neonatal resuscitation techniques in line with established guidelines.

Beyond the initial pilot study, internal consistency of the questionnaire was further evaluated using statistical methods like Cronbach's alpha to ensure the coherence and alignment of questions within each section. The questionnaire administration took place within hospital settings during midwives' break times, minimizing disruption to their routine duties. Analysis of the collected data was performed using SPSS version 25, with frequencies calculated for both independent variables (e.g., age, educational level, and experience) and dependent variables. Chi-square tests were employed for in-depth data analyses, contributing to a robust evaluation of midwives' knowledge and practices in neonatal resuscitation.

### 2.5. Ethical Considerations

The study received written ethical approval from the Sudan Medical Specialization Board Ethical Committee. Written ethical clearance was also obtained from the Khartoum State Ministry of Health, and written permission was obtained from the administrative authorities of the teaching hospitals where the study was conducted. Verbal and written consent was obtained from all participants individually.

### 2.6. Patient and Public Involvement

There was no direct involvement of patients or the public in this study.

## 3. Result

A total of 57 participants were included in the study, with 49.1% belonging to the age group of 51–60 years. All participants were female, and 73.7% were married. 80% of the participants had a one-year diploma level of education, and 22.6% of the midwives worked in Saad Abualilaa and Soba teaching maternity hospitals. 38% of the participants had attended courses for NRPs (Neonatal Resuscitation Programs). 43.8% of the participants had between 21 and 30 years of experience in obstetrics and gynecology, and 49.1% performed neonatal resuscitation on a weekly basis (see Tables 1 and 2 for more details).

Regarding knowledge, 81.7% of the participants mentioned that they prepare for birth by identifying a helper and making an emergency plan. All participants ensured that the delivery area was clean, warm, and well lit. 91.8% washed their hands before touching the baby and helped the mother wash her hands before breastfeeding her baby orally. 82.5% assisted a baby in breathing if necessary, and 96.6% dried the baby thoroughly. 98.2% carried out suction of the fluid from the mouth, while 91.2% began ventilation if the newborn baby was quiet, limp, not crying, and did not respond to steps to stimulate breathing and suction. 66.7% agreed with the recommended rate for ventilating a newborn infant as 20 breaths per minute (see Tables 3 and 4 for more details).

Regarding practice, 50.9% of the participants used all necessary equipment and instruments that were in good working order, ready, available, clean, and sterile. 56.1% did not wear a sterile gown, and 54.4% wore sterile gloves. 71.9% used a clean mask. 98.2% wiped the eyes and face when the head was delivered using sterile cotton material, while 96.5% dried the babies while assessing their breathing. None of the midwives who applied the identification bands included all the necessary details, and none of them placed at least two identification bands on the baby's wrist and mother's wrist. 87.7% of them were administered vitamin K.

Regarding skin-to-skin contact, 29.7% of the participants mentioned that the advantage of skin-to-skin contact is bonding. 91.2% of the participants answered that if the baby is breathing well; they place him/her in skin-to-skin contact with the mother's abdomen and cover the body (see Table 5 for more details).

In terms of resuscitation, 76.4% called for help when they had a baby not breathing well after 30 seconds postdelivery. 63.2% clamped the cord approximately 2-3 minutes after birth or after cessation of cord pulsations. All participants tied the cord firmly about 2 fingers (3-4 cm) from the baby's abdomen and cut the cord 1 cm away from the tie. Only 19.3% thought that cleaning of the airway by suction is necessary, and all started suctioning through the mouth. 94.7% answered that the baby did not need ambo bagging, while 5.3% of babies needed extensive resuscitation using ambo bagging and chest compression. The performance of midwives in ambo bagging and chest compression was poor. 93% thought the baby did not require chest compression, and 5.3% of those who performed ambo bagging performed poorly (see Table 6 for more details).

The study found significant associations between demographic and experiential factors and CPR knowledge and practice. Age, education level, type of courses attended, years of experience, and frequency of performing CPR are all factors that influence CPR knowledge and practice. The findings suggest that educators can improve CPR training and education programs by targeting specific groups based on these factors, as well as by identifying and addressing potential barriers to CPR knowledge and practice (see Table 7 for more details).

## 4. Discussion

The study conducted a comprehensive analysis of the demographic and experiential factors influencing CPR knowledge and practice among midwives, yielding crucial insights that can enhance resuscitation training programs. Of the 57 participants, it was noted that a significant portion (49.1%) belonged to the age group of 51–60 years. The study sheds light on the considerable proportion of midwives in this age range, aligning with findings from other research indicating that more experienced healthcare professionals often exhibit higher proficiency in clinical skills such as neonatal resuscitation [9]. Given that a majority of the participants were in the advanced stages of their careers, their accumulated experience likely contributed to their high proficiency levels measured in this study.

The marital status and educational level also emerged as significant factors. A predominant 73.7% of the participants were married, and 80% had achieved a one-year diploma level of education. This demographic profile may reflect broader trends seen in healthcare, where personal and professional life choices significantly impact career development and specialization [10]. Furthermore, the predominance of diploma-educated individuals highlights the necessity for continuous professional development to ensure that midwives remain updated on the latest protocols and procedures in neonatal care [11].

An important observation from the study is the participation rate in various educational courses. Approximately 38% had attended Neonatal Resuscitation Programs (NRPs), which correlate strongly with improved clinical outcomes in neonatal emergencies. Prior research has established that continuous education, particularly in specialized courses like NRP, significantly enhances the effectiveness of healthcare workers in emergency situations [12, 13]. This underscores the necessity of integrating such programs into regular training schedules for midwives.

The study also brought forth detailed knowledge and practice assessments. An impressive 81.7% of the participants prepared by identifying a helper and making an emergency plan, and 91.8% adhered to hygiene protocols by washing their hands before intervening with the newborn [14]. These practices parallel findings from international studies which emphasize the importance of preparedness and hygiene in reducing neonatal morbidity and mortality [15]. High adherence to these protocols indicates a robust baseline of practical knowledge among the sample population, which is critical in ensuring the success of neonatal resuscitation procedures.

In terms of application, it was observed that 50.9% of the participants used all necessary equipment and 96.5% dried the babies while assessing their breathing. The meticulous adherence to drying and assessing breathing reflects best practice guidelines as recommended by global health authorities such as the World Health Organization (WHO) [16]. However, the discrepancy observed in the usage of sterile gowns and gloves, with 56.1% and 45.6%, respectively, not adhering to the guidelines, indicates areas needing improvement. This inconsistency could potentially compromise the sterility of resuscitation environments and expose newborns to infections.

The data also indicate a gap in the implementation of identification protocols. None of the midwives included all necessary details on the identification bands, and no midwife placed two identification bands on the baby's and mother's wrists [17, 18]. This practice is critical to prevent misidentification, and the current shortfall identified by the study suggests an immediate need for procedural reinforcement in this area.

Importantly, the study's findings on resuscitation practices reveal that a large proportion, 76.4%, did not call for help when a baby was not breathing well after 30 seconds. This can be potentially harmful as prompt action is crucial in neonatal resuscitation to prevent hypoxic damage [19]. The results show that while a number of participants have high theoretical knowledge, there may be gaps in practical application, especially under high-pressure conditions.

The observed low rate of good performance in ambu bagging and chest compressions (only 5.3% reflecting good performance) is particularly concerning. Previous studies have shown that hands-on practice and simulation-based training are essential in improving these skills among healthcare providers [20]. The findings here reiterate the need for more frequent and rigorous practical training sessions to enhance the proficiency of midwives in performing effective resuscitation measures [21].

Finally, the study draws significant attention to the associations between demographic factors, years of experience, frequency of CPR performance, and levels of knowledge and practice. These findings suggest the necessity for targeted interventions in educational programs, highlighting the importance of continuous learning and practice tailored to the specific needs identified within different demographic groups. Regular assessment and refresher courses may bridge the knowledge-practice gap observed [22, 23].

The current situation in Sudan regarding CPR, particularly neonatal resuscitation, highlights the need for targeted training programs to enhance midwives' proficiency in life-saving techniques. The study's implications emphasize the importance of continuous professional development and hands-on training to improve neonatal resuscitation outcomes [24]. Conducting research in specific hospital settings in Khartoum allows for a focused examination of practices and knowledge among midwives in urban healthcare settings, offering insights that can be utilized to tailor interventions for improved maternal and child healthcare.

In conclusion, this study provides valuable insights into the factors influencing CPR knowledge and practice among midwives and offers actionable information to enhance training programs. Emphasizing continuous professional development, adherence to best practices, and the integration of frequent hands-on training can significantly improve neonatal resuscitation outcomes. These improvements will contribute towards better neonatal care and lower infant morbidity and mortality rates, aligning with global healthcare goals.

### 4.1. Study Limitations

One limitation of the study is the small sample size of 57 participants, which may restrict the generalizability of the findings to a larger population of midwives. In addition, the study's focus on specific hospitals in Khartoum limits the generalizability of the results to other healthcare settings. The reliance on self-reporting through questionnaires and interviews may introduce response bias, and the lack of a comparative analysis and limited scope of assessment restrict the ability to assess the effectiveness of interventions or benchmark against other healthcare professionals. These limitations were identified and acknowledged to provide a transparent and comprehensive interpretation of the study findings. Nonetheless, further research with larger sample sizes and more diverse participant groups is warranted to address these limitations and enhance the validity and generalizability of the findings.

## 5. Conclusion

In conclusion, this study provides valuable insights into the knowledge and practices of midwives in Sudan regarding neonatal resuscitation. Although many midwives displayed adequate knowledge and adherence to certain practices, there remains room for improvement in areas such as the use of necessary equipment, identification band details, and ambo bagging and chest compression skills. The study also highlights the influence of demographic and experiential factors on CPR knowledge and practice.

## 6. Recommendations

We recommend the development of targeted CPR training and education programs that address demographic and experiential factors to improve the overall quality of neonatal resuscitation in Sudan. In addition, efforts should be made to address potential barriers to CPR knowledge and practice, such as limited resources or lack of support. By implementing these recommendations, we can enhance the outcomes for newborns requiring resuscitation and ultimately improve the quality of maternal and child healthcare in Sudan.

## Figures and Tables

**Table 1:** Demographic characteristics of the participants, *N* = 57.

Characteristics	Groups	Frequency	Percentage (%)
Age	30–40 years old	5	8.90
	41–50 years old	16	28
	51–60 years old	28	49.10
	More than 60 years old	8	14

Marital status	Single	0	0
	Married	42	73.70
	Widow	11	19.30
	Divorced	4	7

Educational level	Pregraduate one-year diploma	45	80
	Bachelor of nursing and midwifery	5	8.30
	Three-year diploma	4	6.70
	Bachelor of midwifery specialist	3	5

Years of experience	Less than 10 years	9	15.80
	10–20 years	14	24.60
	21–30 years	25	43.80
	31 years	9	15.80

**Table 2:** Type of courses attended by studied participants, *N* = 57.

Type of courses attended	Frequency	Percentage (%)
Helping baby to breath	29	50.8
Essential steps in management of obstetric emergency (ESMOE)	27	47.4
NRP	46	80.7
Pediatric life support	19	33.3

**Table 3:** Knowledge assessment (part A) distribution among studied participants, *N* = 57.

Knowledge assessment (part A)	Frequency	Percentage (%)
To prepare for a birth		
You identify a helper and make an emergency plan	49	81.7
You ask everyone but the mother to leave the area	7	11.7
You prepare equipment only when you need it	2	3.3
You do not need a helper	2	3.3
To prepare the area for delivery		
Open all the doors and windows to get fresh air	0	0.0
A clean space for the baby will not be required	0	0.0
Make sure the area is clean, warm, and well lighted	57	100.0
Keep the room temperature cold	0	0.0
What should you do to keep the baby clean?		
Wash your hands before touching the baby and help mother wash her hands before breastfeeding	52	91.2
Reuse the suction device before cleaning	1	1.8
Keep the umbilical cord tightly covered	4	7.0
Do not touch the baby	0	0.0
What should you do in the golden minute?		
Bathe the baby	2	3.5
Deliver the placenta	4	7.0
Evaluate the heart rate	4	7.0
Help a baby breathe if necessary	47	82.5
A baby is quiet, limp, and not breathing at birth. What should you do?		
Dry the baby thoroughly	57	96.6
Shake the baby	0	0.0
Throw cold water on the face	0	0.0
Hold the baby upside down	2	3.4
If the baby is still not breath, what is your next step?		
Hold the baby upside down	1	1.8
Suction the fluid from mouth	56	98.2
Throw cold water on the face	0	0.0
Shake the baby	0	0.0
Newborn baby is quiet, limp, and not crying. The baby does not respond to steps to stimulate breathing and suction. What should you do next?		
Slap the baby's back	3	5.3
Begin ventilation	52	91.2
Squeeze the baby's ribs	2	3.5
Throw water on the face	0	0.0
Which of the following rates is recommended for ventilating a newborn infant?		
20 breaths per minute	38	66.7
40 breaths per minute	9	15.8
80 breaths per minute	8	14.0
100 breaths per minute	2	3.5

**Table 4:** Knowledge assessment (part B) distribution among studied participants, *N* = 57.

Knowledge assessment (part B)	Frequency	Percentage (%)
Advantage of skin-to-skin contact		
Prevent hypothermia	50	87.7
Help baby stay warm	48	84.2
Bonding	57	100
Help expel placenta and uterine contraction	37	64.9
About providing eye ointment after deliveries		
Prevent eye infection	46	80.7
Prevent blindness	13	22.8
Not important	11	19.2
In order to have an effective ventilation with bag and mask		
The mask should cover the eyes	3	5.3
Air should escape between the mask and face	2	3.5
Squeeze the bag to produce gentle movement of the chest	55	96.5
Squeeze the bag to give 80–100 breaths per minute	11	19.3
You can stop ventilation if:		
Baby is blue and limp	2	3.5
Baby's heart rate is 80 per minute	14	24.6
Baby's heart rate is 120 per minute and the chest is not moving	8	14
Baby's heart rate is 120 per minute and the baby is breathing or crying	54	94.7
In newborn babies who do not require positive-pressure ventilation, the cord should be clamped earlier than one minute after birth		
True	22	38.5
False	35	61.5
Vitamin K should be given to all newborn babies		
True	55	96.5
False	2	3.5

**Table 5:** Practice assessment (part A) distribution among study participants, *N* = 57.

Practice assessment (part A)	Frequency	Percentage (%)
All needed equipment and instruments (warmer, ambo bag, suction device, sterile bulb, and oxygen) in a good working order, ready, available, and clean/sterile		
Yes	29	50.9
No	28	49.1
Wearing sterile gown		
Yes	25	43.9
No	32	56.1
Wearing sterile gloves		
Yes	31	54.4
No	26	45.6
Clean mask		
Yes	41	71.9
No	16	28.1
Wipes the eyes and face when the head is delivered using sterile cotton material		
Yes	56	98.2
No	1	1.8
After full delivery of the baby, dries the baby while assessing the baby's breathing		
Yes	55	96.5
No	2	3.5
They put identification band		
Yes	22	38.6
No	35	61.4
If yes, the identification band putting before cutting cord		
Yes	0	0.0
No	22	100%
The identification band includes the mother's full name, hospital admission, sex of the infant, and date and time of delivery		
Yes	0	0.0
No	22	100%
Put at least two identification bands on baby's wriest and mother's wrist		
Yes	0	0.0
No	22	100
Not applicable	35	61.4
Vitamin K administered	0	0.0
Yes	50	87.7
No	7	12.3
Total	57	100.0

**Table 6:** Practice assessment (part B) distribution among studied participants, *N* = 57.

Practice assessment (part B)	Frequency	Percentage (%)
If the baby breathing well, place him/her in skin-to-skin contact on the mother's abdomen and cover the body		
Yes	46	80.7
No	11	9.3
If baby not breathing well after 30 second after delivery		
Call for help	7	63.6
Start resuscitations	4	36.4
Clamps the cord approximately 2-3 minutes after the birth or after cessation of cord pulsations		
Yes	36	63.2
No	21	36.8
Ties the cord firmly about 2 fingers (3-4 cm) from the baby's abdomen and cuts the cord 1 cm away from the tie		
Yes	57	100.0
No	0	0.0
Cleaning of airway by suction was needed?		
Yes	11	19.3
No	46	80.7
If yes, which was firstly sucked		
Mouth	11	100
Nose	0	0.0
Did the baby need ambo bagging?		
Yes	3	5.3
No	54	94.7
If yes, how it performed		
Good performance	0	0.0
Poor performance	3	100
Did baby require chest compression?		
Yes	4	7.0
No	53	93.0
If yes, how it performed		
Good performance	0	0.0
Poor performance	4	100

**Table 7:** Chi-square test and *p* value distribution among studied participants, *N* = 57.

1st variable	2nd variable	*p* value
Age	Level of knowledge	0.01
Educational level	Level of knowledge	0.00
Type of courses attended	Level of knowledge	0.01
Years of experience	Level of knowledge	0.00
Frequency of CPR performance	Level of knowledge	0.03

Age	Level of practice	0.00
Educational level	Level of practice	0.07
Type of courses attended	Level of practice	0.09
Years of experience	Level of practice	0.00
Frequency of CPR performance	Level of practice	0.00

## Data Availability

The datasets used and/or analyzed during the study are available from the corresponding author upon reasonable request.
